# Study on the effects of urea addition on the fermentation quality, nitrogen metabolism, microbial community, and metabolic characteristics of cotton strawlage

**DOI:** 10.3389/fmicb.2025.1610850

**Published:** 2025-06-23

**Authors:** Shuaibin Zhou, Zhenxin Zhu, Xiaoping Chen, Qiuju Pu, Shuai Liu, Yuheng Luo, Yongbin Shao, Yaoqiang Sun, Xinwen Sun, Cuiling Shang, Wen Shen, Dengke Hua, Xinfeng Wang

**Affiliations:** ^1^Laboratory of Forage Comprehensive Utilization, College of Animal Science and Technology, Shihezi University, Shihezi, China; ^2^Tumushuke Tuxing Animal Husbandry Co., Ltd., Tumushuke, China; ^3^Nileke County's Animal Husbandry and Veterinary Development Center of Yili Prefecture, Nileke, China; ^4^Animal Nutrition Institute, Sichuan Agricultural University, Chengdu, China

**Keywords:** cotton strawlage, urea, nitrogen metabolism, microbial community, metabolic characteristics

## Abstract

**Background:**

Cotton straw is a widely available agricultural by-product in Xinjiang, China, but its utilization as livestock feed is hindered by challenges such as high fiber content and the presence of toxic free gossypol, which can negatively impact animal health and feed digestibility. Urea addition in feed fermentation has shown potential in improving feed quality by providing a nitrogen source for microorganisms, promoting fiber degradation, and increasing the content of crude protein. However, the optimal urea addition level for cotton straw fermentation remains unclear. The objective of this study was to explore the effects of urea addition on the fermentation quality, nitrogen fractions, bacterial community, and metabolic characteristics of cotton strawlage. The experiment included a control group with 0 % urea added (U0), a 0.1% urea group (U1, 1 g/kg fresh matter), a 0.2% urea group (U2, 2 g/kg fresh matter), and a 0.4% urea group (U3 4 g/kg fresh matter). After 45 days of fermentation, samples of the strawlage were collected and analyzed for their chemical composition, fermentation quality, nitrogen fractions, bacterial community, and metabolic characteristics.

**Result:**

Compared to the U0 group, the U3 group significantly increased total nitrogen (TN), non-protein nitrogen (NPN), acetic acid, and propionic acid contents by 17.93%, 0.08%, 13.26%, and 0.01%, respectively, while significantly reducing free gossypol and non-structural nitrogen (NSN) levels by 48.50% and 14.22%, respectively, thereby optimizing the protein composition of cotton straw. Furthermore, the U1 and U2 groups exhibited significant downregulation of metabolites associated with amino acid and energy metabolism pathways, such as L-arginine, L-proline, L-ornithine, and 2-Oxoglutaric acid, compared to the U0 group.

**Conclusion:**

The addition of urea significantly altered the fermentation quality, nitrogen fraction composition, microbial community composition, and metabolic profiles of cotton straw.

## 1 Introduction

Cotton serves as a globally significant source of natural textile fibers and oil crops (Lee et al., 2007; Zaidi et al., 2020; Jan et al., 2022). Xinjiang, China, has emerged as the country's leading cotton-producing region, thanks to its unique geographical and climatic conditions, including a vast land area, prolonged sunshine duration, an arid climate, and marked diurnal temperature variations. According to statistical data from the National Bureau of Statistics Announcement on Cotton Production, 2022, Xinjiang's cotton planting area reached 2.5 million hectares, yielding 5.391 million tons of cotton, which accounted for over 90% of China's total production (National Bureau of Statistics Announcement on Cotton Production, 2022). Based on the straw-to-grain ratio model proposed by Cai et al. (2024) (assuming a cotton straw-to-cotton ratio of 5:1), the theoretical annual cotton straw production in Xinjiang was ~26.955 million tons. The nutritional composition varies significantly among different cotton straw tissues (roots, stems, leaves) (Zhang et al., 2018), with whole-plant averages of 6.41% crude protein, 34.76% cellulose, 15.58% hemicellulose, 10.29% lignin, and 23.34 MJ/kg of gross energy. Leaves and boll shells, which have relatively higher nutrient contents, show potential as ruminant feed resources (Gong, 2007; Zhang et al., 2018). However, their feed utilization is severely constrained by high crude fiber content (with neutral detergent fiber at 65.42%) and toxic free gossypol levels (ranging from 0.02% to 0.2%).

Urea, an odorless, tasteless, deliquescent white crystalline granular substance with a high nitrogen content of 46.6% and low cost, is widely used as a feed additive in ruminant husbandry. Studies have demonstrated that urea supplementation can significantly reduce neutral detergent fiber content in silage, while simultaneously increasing organic acid content and aerobic stability (Fu, 2014; Tan et al., 2014). This technology not only effectively mitigates the risks of ammonia poisoning associated with direct urea feeding but it also enhances the crude protein content through microbial transformation, improving feed intake and dry matter digestibility (Cairang et al., 2014; Costa et al., 2020).

The Cornell Net Carbohydrate and Protein System (CNCPS), developed by Cornell University, provides a precise framework for evaluating feed nutritional value by classifying nutrients based on animal digestion characteristics (Wang X. G. et al., 2024). Although previous research confirmed urea's role in increasing crude protein content in fermented feeds (Costa et al., 2020), a systematic understanding of its effects on nitrogen fraction composition and microbial regulatory mechanisms remains limited. This study applied a simplified nitrogen fractionation approach within the CNCPS framework, combined with 16S rRNA sequencing and untargeted metabolomics, to investigate the effects of urea supplementation on nitrogen nutrition transformation in cotton straw. The findings aim to provide theoretical support for the efficient utilization of cotton straw resources.

## 2 Materials and methods

### 2.1 Cotton straw preparation

Cotton straw (the variety was Xinluzao No.84) were harvested on October 3, 2023, in Liangzhouhu Town, Manas County, Changji Prefecture, Xinjiang (43°28′-45°38′N, 85°34′-86°43′E). The cotton straw used for ensiling strawlage were chopped into 1–2 cm pieces using a forage crusher. The compound fermented bacterial liquid and molasses were purchased from Shihezi Dongke Animal Husbandry Technology Co., Ltd., China.

A single-factor experimental design was employed in this study, with four treatment groups established: (1) U0 (no urea added); (2) U1 (0.1% urea added); (3) U2 (0.2% urea added); (4) U3 (0.4% urea added). For each treatment, 1% of microbial liquid (*Lactobacillus plantarum* ≥ 1.5 × 106 CFU/mL, *Candida utilis* ≥ 3.0 × 10^5^ CFU/mL, *Saccharomyces cerevisiae* ≥ 1.0 × 106 CFU/mL, *Candida tropicalis* ≥ 1.2 × 105 CFU/mL, *Bacillus subtilis* ≥ 2.0 × 106 CFU/mL, *Geotrichum candidum* ≥ 1.0 × 104 CFU/mL, *D-FGP strain* ≥ 5 × 104 CFU/mL, *D-CF strain* ≥ 3.0 × 105 CFU/mL) were added as inoculum and 5% molasses (based on fresh weight, FW) were added as energy source for microbes. Then the distilled water was added to adjust the moisture content to ~65% (FW). After mixing thoroughly, 500 g of the treated straw was packed into laboratory sterile plastic bags with release valves, vacuum-sealed, and stored at room temperature for 45 days, according to Xu's (2022) study.

### 2.2 Chemical composition analyses

Some fresh samples of fermented cotton strawlage after 45 days were collected for determining the enzymes activity and chemical composition. Firstly, urease, glutamate synthase, glutamine synthetase, and glutamate dehydrogenase assay kits (all purchased from Jiangsu Jingmei Biotechnology Co., Ltd., China) were used to determine the activities of these enzymes. Appropriate samples from each treatment group were placed into the oven (LBAO-250; Shanghai Sidike Instrument and Equipment Co., Ltd., Shanghai, China) and dried at 65°C for 48 h. Afterward, they were removed from the oven and allowed to absorb moisture for 24 h to make the samples reach equilibrium with the humidity of the surrounding environment to ensure the accuracy of the dry matter determination (Wang et al., 2025). Subsequently, the samples were crushed using a crusher (FS200; Guangzhou Bomin Electromechanical Equipment Co., Ltd., Guangzhou, China) and sieved through 1.0 mm and 2.0 mm mesh, respectively. The dry matter (DM), crude protein (CP), and ether extract (EE) were determined by the AOAC method (AOAC, 2006). The neutral detergent fiber (NDF) and acidic detergent fiber (ADF) were analyzed according to the method described in the previous report (Van Soest et al., 1991). The WSC concentration was determined by the sulfuric acid-anthrone colorimetric method (Yemm and Willis, 1954). Nitrogen distribution fractions were analyzed according to the method described in the previous report (Liu, 2024). The free gossypol content was determined in accordance with GB/T 13086-2020 (National Feed Industry Standardization Technical Committee, 2020).

### 2.3 Fermentation quality analysis

The fermentation quality was assessed after 45 days of fermentation. A sample of 20 g from each treatment was mixed with 180 mL of distilled water. After sealing with parafilm, the mixture was stored in a refrigerator at 4 °C for 24 h for extraction. The extract was then filtered through four layers of gauze and qualitative filter paper. The pH of the filtered solution was determined using a digital pH meter (PHBJ- 260F; Shanghai INESA Scientific Instrument Co., Ltd., China). The ammonia nitrogen concentration was measured using the phenol-sodium hypochlorite method (Broderick and Kang, 1980). Organic acids were quantified using high-performance liquid chromatography (1,260 Infinity II; Agilent Technologies, Waldbronn, Germany) as described by Xie et al. (2023).

### 2.4 DNA extraction and amplification

Microbial DNA was extracted from samples using the E.Z.N.A.^®^ Soil DNA Kit (Omega Bio-tek, Norcross, GA, U.S.) according to manufacturer's protocols. The V4-V5 region of the bacteria 16S ribosomal RNA gene was amplified by PCR under the following conditions: 95°C for 2 min, followed by 25 cycles at 95°C for 30 s, 55°C for 30 s, and 72°C for 30 s, and a final extension at 72°C for 5 min. The primers used were 515F 5′-barcode- GTGCCAGCMGCCGCGG-3′ and 907R 5′-CCGTCAATTCMTTTRAGTTT-3′, where barcode is an eight-base sequence unique to each sample. PCR reactions were performed in triplicate 20 μL mixture containing 4 μL of 5 × FastPfu Buffer, 2 μL of 2.5 mM dNTPs, 0.8 μL of each primer (5 μM), 0.4 μL of FastPfu Polymerase, and 10 ng of template DNA. Amplicons were extracted from 2% agarose gels and purified using the AxyPrep DNA Gel Extraction Kit (Axygen Biosciences, Union City, CA, U.S.) according to the manufacturer's instructions.

### 2.5 Library construction and sequencing

SMRTbell libraries were prepared from the amplified DNA by blunt-ligation according to the manufacturer's instructions with SMRTbell prep kit 3.0 (Pacific Biosciences, PN: 102-182-700). Purified SMRTbell libraries from the pooled and barcoded samples were sequenced on a single PacBio Sequel IIe cell. All amplicon sequencing was performed by Shanghai Biozeron Biotechnology Co. Ltd (Shanghai, China).

### 2.6 Processing of sequencing data

PacBio raw reads were processed using the SMRT Link Analysis software version 11.0 to obtain demultiplexed circular consensus sequence (CCS) reads with the following settings: minimum number of passes = 3, minimum predicted accuracy = 0.99. Raw reads were processed through SMRT Portal to filter sequences for length (>1000 or <1800 bp) and quality. Sequences were further filtered by removing barcode and primer sequences with lima pipeline (Pacific Biosciences demultiplexing barcoded software, https://lima.how/).

### 2.7 Operational units (OTUs) generation

OTUs were clustered with 98.65% (Edgar, 2018; Johnson et al., 2019; Gao et al., 2022) similarity cutoff using UPARSE (Edgar, 2013) (version 10, http://drive5.com/uparse/). The phylogenetic affiliation of each 16S rRNA gene sequence was analyzed by uclust algorithm (Edgar, 2010) (https://github.com/topics/uclust) against the Silva (SSU138.2) (Quast et al., 2012) 16S rRNA database (http://www.arb-silva.de) using confidence threshold of 80% (Kim and Chun, 2014). After ASVs generation, the analysis of the full-length 16S rDNA data were operated by CFViSA platform, which contained microbiome analysis pipeline and nearly 80 analysis tools spanning simple sequence processing, visualization, and statistics available for the amplicon sequencing data.

### 2.8 LC-MS analysis

The LC-MS analysis was performed to identify metabolites, using an Ultimate 3000LC-Q-Exactive instrument (Thermo, CA, USA) equipped with a Hyper gold C18 column (100 mm by 2.1 mm, 1.9 μm; Thermo).The column temperature was maintained at 40°C. The mobile phase consisted of mobile phase A (water plus 5% [vol/vol] acetonitrile and 0.1% [vol/vol] formic acid) and mobile phase B (acetonitrile plus 0.1% [vol/vol] formic acid) at a flow rate of 0.3 mL/min. The elution procedure was as follows: 5% mobile phase B from 0 to 1 min, 5% to 95% mobile phase B from 1 to 11 min, and 95% to 5% mobile phase B from 11 to 19.5 min. The injection volume was 10 μL, and the autosampler was maintained at 4°C. The mass spectrometric settings for positive/negative-ion modes were as follows: a heater temperature of 300°C, a sheath gas flow rate of 45 arb, an auxiliary gas flow rate of 15 arb, a sweep gas flow rate of 1 arb, a spray voltage of 3.0 kV/3.2 kV, a capillary temperature of 350°C, and an S-ens radio frequency level of 30%/60%, respectively.

The raw data were analyzed using feature extraction and preprocessing by Compound Discoverer 2.0 software (Thermo Fisher Scientific Inc, USA). lon peak data present in <50% of the samples were removed. The main parameters were set as follows: an intensity threshold of 300,000, an *m/z* range of 70 to 1,050, an *m/z* width of 5 ppm, a frame time width of 0.2 min, and retention time start and end values of 0.01 and 19.5 min, respectively. The data were normalized according to the internal label and post-edited using Excel 2010 software. The online Human Metabolome Database (https://hmdb.ca) and KEGG database (https:/www.genome.jp/kegg/) were used to identify metabolites by aligning the molecular mass data. The metabolites were reported only when the difference between the theoretical mass and the observed mass was <20 ppm and further validated by isotopic distribution measurement. Principal component analysis (PCA), orthogonal partial least squares discriminant analysis (OPLS-DA), and loading plots were conducted using SlMCA-P software (version 13.0: Umetrics, Umea, Sweden). The OPLS-DA models were validated based on the variation interpretation (R^2^Y) and predictability (Q^2^) of the model in cross-validation and permutation tests with 200 iterations. Differential metabolites were identified according to the VIP obtained from the OPLS-DA model and statistical analysis (VIP score >1 and *P-value* <0.05). When metabolites were identified in both positive and negative ion modes, data from the mode with the lower *P-value* were retained.

### 2.9 Correlation between bacterial community and enzymes and nitrogen fractions

The correlation between the affected bacterial genera with a relative abundance >0.02% and the enzymes, as well as the correlation between these affected bacterial genera and nitrogen fractions, was assessed separately by Pearson's correlation analysis in Origin (version 2024). These correlations were visualized using the app of Correlation plot (version v1.31).

One-way analysis of variance (ANOVA) was conducted using IBM SPSS Statistics software to analyze the data on affected bacteria due to urea addition. In addition, regression analysis of chemical composition and fermentation quality was conducted using curve estimation with IBM SPSS statistics software.

### 2.10 Statistical analysis

All microbiota data were submitted to the NCB (National Centre of Biotechnology Information Bethesda, Maryland, USA) Sequence Read Archive (SRA) database (accession number SUB15181778).

## 3 Results

### 3.1 Chemical composition of cotton straw before and after fermentation

The chemical composition of cotton straw before being fermented is presented in Table 1. The DM content was 89.60% fresh material. CP, NDF, ADF, EE, Ash, WSC, and FG contents were 8.38%, 54.11%, 40.08%, 5.36%, 8.13%, 2.14%, and 269.09 mg/kg, respectively.

**Table 1:** Chemical composition of cotton straw before fermentation.

**Item^a^**	**Content**	**SEM^b^**
DM	89.60	0.532
CP (% DM)	8.38	0.228
NDF (% DM)	54.11	0.527
ADF (% DM)	40.08	0.291
EE (% DM)	5.36	0.138
Ash (% DM)	8.13	0.072
WSC (% DM)	2.14	0.177
FG (mg/kg)	269.09	15.600

^a^DM, drymatter; CP, crude protein; NDF, neutral detergent fiber; ADF, acid detergent fiber; EE, ether extract; WSC, water-soluble carbohydrates; FG, free gossypol.

^b^SEM, standard error of the mean.

The chemical compositions of fermented cotton strawlage are presented in Table 2. With the exception of NDF, all other indices presented in the table were significantly influenced by the increasing addition of urea. DM and ADF contents initially decreased and then increased, following a cubic trend, peaking at U3 (*P* = 0.035 for DM, and *P* = 0.031 for ADF). As expected, CP content increased in a linear, quadratic, and cubic manner with the addition of urea. In contrast, the FG content decreased linearly, quadratically, and cubically as urea was added. A quadratic and cubic decline of EE and WSC contents was observed with increasing urea addition. No significant differences were noted in NDF and ash contents with varying levels of urea addition; however, the increased addition of urea exhibited a cubic trend effect on ash content.

**Table 2:** The chemical compositions of cotton strawlage after 45 days of fermentation with varying levels of urea (%DM).

**Item^1^**	**U0**	**U1**	**U2**	**U3**	**SEM^2^**	***P*** **-** ***value***		
						**Lin^3^**	**Quad^4^**	**Cub^5^**
DM	34.17^ab^	34.69^a^	33.17^b^	35.31^a^	0.287	0.479	0.299	0.035
CP	7.33^c^	7.43^c^	8.14^b^	9.30^a^	0.208	<0.001	<0.001	<0.001
NDF	42.88	42.65	42.05	42.79	0.548	0.870	0.905	0.961
ADF	27.75^a^	28.05^a^	25.67^b^	28.29^a^	0.377	0.830	0.316	0.031
EE	7.98^a^	6.98^b^	6.85^b^	7.58^a^	0.141	0.305	<0.001	0.002
Ash	8.97^ab^	8.70^b^	8.94^ab^	9.02^a^	0.498	0.419	0.158	0.105
FG	205.59^a^	185.69^b^	155.69^c^	105.87^d^	9.724	<0.001	<0.001	<0.001
WSC	1.36^a^	1.32^b^	1.30^bc^	1.27^c^	0.103	<0.001	<0.001	0.002

^a − c^Values with different superscripts within the same row differ significantly (*P* <0.05).

1DM, dry matter; CP, crude protein; NDF, neutral detergent fiber; ADF, acid detergent fiber; EE, ether extract; WSC, water-soluble carbohydrates; U0, without urea; U1, the addition of 0.1% urea; U2, the addition of 0.2% urea; U3, the addition of 0.4% urea.

^2^SEM, standard error of the mean.

^3^Lin, linear effect.

^4^Quad, quadratic effect.

^5^Cub, cubic effect.

### 3.2 Effect of urea on fermentation quality of cotton strawlage

Fermentation characteristics of cotton strawlage are presented in Table 3. The addition of urea resulted in linear, quadratic, and cubic increases in AA (acetic acid) and PA (propionic acid) contents, reaching peaks of 14.35 μg/mL (U3) and 2.43 μg/mL (U2 and U3), respectively. However, the differences in pH and LA (lactic acid) content between U0 and the other treatments with varying levels of urea were not significant. BA was not detected in any of the groups.

**Table 3:** Fermentation characteristics of cotton strawlage after 45 days of fermentation with varying levels of urea.

**Item^1^**	**U0**	**U1**	**U2**	**U3**	**SEM^2^**	***P*** **-** ***value***		
						**Lin^3^**	**Quad^4^**	**Cub^5^**
pH	4.64	4.67	4.69	4.64	0.025	1.000	0.722	0.880
LA (mmol/mL)	5.16	5.44	5.33	5.52	0.07	0.127	0.308	0.327
AA (μg/mL)	12.67^bc^	11.69^c^	13.59^ab^	14.35^a^	0.320	0.010	0.009	0.005
PA (μg/mL)	2.41^b^	2.40^b^	2.43^a^	2.43^a^	0.004	<0.001	0.005	<0.001
BA (μg/mL)	ND^6^	ND	ND	ND	–	–	–	–

^a − c^Values with different superscripts within the same row differ significantly (P <0.05).

^1^pH, pH-value; LA, lactic acid; AA, acetic acid; PA, propionic acid.

^2^SEM, standard error of the mean.

^3^Lin, linear effect.

^4^Quad, quadratic effect.

^5^Cub, cubic effect.

^6^ND indicates that no data has been detected.

### 3.3 Effect of urea on nitrogen distribution fractions and amino acid profiles of cotton strawlage

Nitrogen distribution fractions of cotton strawlage after 45-day fermentation are presented in Table 4. It was obvious that urea addition had both quadratic and cubic effects on the contents of TN, NPN, PN, FAAN, and NH_3_-N. Notably, there was a marked linear, quadratic, and cubic increase in FAAN and NH_3_-N. Compared to U0, TN increased significantly from 1.41% to 1.71%, NPN increased from 52.35% TN to 59.17%, and PN increased from 43.24% TN to 47.90% as urea addition rose from 0.1% to 0.4%. However, upon completion of the fermentation process, no differences in NH_3_-N content were observed among the treatment groups with increasing urea additions, all of which were higher than U0. Furthermore, FAAN content initially increased and then decreased quadratically and cubically (*P* = 0.041, *P* = 0.013, respectively), reaching its highest point with the addition of 0.1% urea.

**Table 4:** Nitrogen distribution fractions of cotton strawlage after 45 days of fermentation with varying levels of urea.

**Item^1^**	**U0**	**U1**	**U2**	**U3**	**SEM^2^**	***P*** **-** ***value***		
						**Lin** ^3^	**Quad** ^4^	**Cub** ^5^
TN, %DM	1.45^c^	1.41^c^	1.56^b^	1.71^a^	0.033	<0.001	<0.001	<0.001
TPN, %TN	38.03^a^	35.23^ab^	35.37^ab^	32.57^b^	0.720	0.006	0.028	<0.001
NSN, %TN	9.63^a^	9.93^a^	8.22^b^	8.26^b^	0.234	0.002	0.009	<0.001
NPN, %TN	52.35^c^	54.84^bc^	56.41^ab^	59.17^a^	0.806	<0.001	0.002	0.006
PN, %TN	43.24^b^	41.28^b^	44.90^ab^	47.90^a^	0.816	0.010	0.006	0.011
FAAN, %TN	5.83^b^	8.80^a^	7.04^b^	6.98^b^	0.322	0.574	0.041	0.001
NH_3_-N, %TN	3.28^b^	4.77^a^	4.47^a^	4.29^a^	0.190	0.110	0.012	0.013

^a − c^Values with different superscripts within the same row differ significantly (P <0.05).

^1^TN, total nitrogen; TPN, soluble true protein nitrogen; NSN, acid detergent insoluble protein nitrogen; NPN, non-protein nitrogen; PN, peptide nitrogen; FAAN, free amino acid nitrogen; NH3-N, ammonia nitrogen.

^2^SEM, standard error of the mean.

^3^Lin, linear effect.

^4^Quad, quadratic effect.

^5^Cub, cubic effect.

The amino acid profiles of cotton straw are presented in Table 5. Aspartic acid (Asp), threonine (Thr), serine (Ser), leucine (Leu), arginine (Arg), and proline (Pro) exhibited significant linear, quadratic, and cubic decreases in concentration with increased urea supplementation. Glycine (Gly), valine (Val), phenylalanine (Phe), and lysine (Lys) concentrations showed significant reductions in both linear and quadratic patterns. Conversely, tyrosine (Tyr) and histidine (His) demonstrated significant quadratic and cubic responses, while Glutamic acid (Glu) levels decreased significantly in both linear and cubic manners. No differences were observed in isoleucine (Ile) content across all treatment groups.

**Table 5:** Amino acids contents of cotton straw after 45 days of fermentation with varying levels of urea.

**Item^1^**	**U0**	**U1**	**U2**	**U3**	**SEM^2^**	***P*** **-** ***value***		
						**Lin^3^**	**Quad^4^**	**Cub^5^**
Asp	0.840^b^	1.219^a^	0.742^c^	0.566^d^	0.062	0.013	<0.001	<0.001
Thr	0.123^b^	0.136^a^	0.117^c^	0.111^d^	0.002	0.006	<0.001	<0.001
Ser	0.130^a^	0.124^ab^	0.119b^c^	0.113^c^	0.002	<0.001	<0.001	0.002
Glu	0.592^b^	0.746^a^	0.489^d^	0.527^c^	0.026	0.043	0.063	<0.001
Gly	0.139^b^	0.142^b^	0.144^b^	0.179^a^	0.006	0.030	0.038	0.079
Ala	0.179^b^	0.206^a^	0.152^d^	0.167^c^	0.005	0.054	0.138	<0.001
Val	0.116^b^	0.118^b^	0.118^b^	0.147^a^	0.005	0.042	0.041	0.081
Ile	0.100^a^	0.100^a^	0.100^a^	0.121^a^	0.004	0.073	0.073	0.140
Leu	0.191^a^	0.178^b^	0.18^b^	0.163^c^	0.003	<0.001	<0.001	<0.001
Tyr	0.125^c^	0.174^a^	0.146^b^	0.101^d^	0.007	0.117	<0.001	<0.001
Phe	0.114^b^	0.114^b^	0.116^b^	0.142^a^	0.005	0.031	0.029	0.066
Lys	0.150^b^	0.153^b^	0.153^b^	0.192^a^	0.007	0.030	0.031	0.059
His	0.048^c^	0.068^a^	0.044^d^	0.058^b^	0.002	0.832	0.010	<0.001
Arg	0.114^a^	0.034^b^	0.034^b^	0.013^c^	0.010	<0.001	<0.001	<0.001
Pro	0.490^a^	0.501^a^	0.417^b^	0.393^c^	0.012	<0.001	<0.001	<0.001

^a − d^Values with different superscripts within the same row differ significantly (*P* < 0.05). U0, without urea; U1, the addition of 0.1% urea; U2, the addition of 0.2% urea; U3, the addition of 0.4% urea.

^2^SEM, standard error of the mean.

^3^Lin, linear effect.

^4^Quad, quadratic effect.

^5^Cub, cubic effect.

### 3.4 Effect of urea on urease and ammonia assimilation enzyme activities of cotton strawlage

Urease, glutamate synthase (GS), glutamine-oxoglutarate aminotransferase (GOGAT), and glutamate dehydrogenase activities (GDH) are presented in Table 6. Urease activity increased linearly, quadratically, and cubically with the addition of urea, reaching its lowest point at 6,764.81 U/g with the addition of 0.4% urea. GS activity increased by 20% in U2 compared to the U0, and this increase followed a cubic trend with rising urea levels. GDH activity initially decreased and then increased significantly, exhibiting quadratic and cubic responses to urea concentrations ranging from 0.1% to 0.4%. However, no significant differences in GOGAT activity were detected among the treatment groups.

**Table 6:** Urease, GOGAT, GS, and GDH concentrations in cotton strawlage after 45 days of fermentation with varying levels of urea (U/g).

**Item^1^**	**U0**	**U1**	**U2**	**U3**	**SEM^2^**	***P*** **-** ***value***		
						**Lin^3^**	**Quad^4^**	**Cub^5^**
Urease	7561.24^a^	7590.09^a^	7728.27^a^	6764.81^b^	111.85	0.018	<0.001	<0.001
GOGAT	2.45	2.73	2.56	2.22	0.086	0.274	0.096	0.199
GS	0.30^b^	0.29^b^	0.36^a^	0.26^b^	0.013	0.665	0.187	0.012
GDH	0.118^b^	0.112^c^	0.103^d^	0.133^a^	0.003	0.185	<0.001	<0.001

^a − c^Values with different superscripts within the same row differ significantly (*P* < 0.05).

^1^Urease, urease activity; GOGAT, glutamate synthase activity; GS, glutamine synthetase activity; GDH, glutamate dehydrogenase activity; CON, without urea; U1, the addition of 0.1% urea; U2, the addition of 0.2% urea; U3, the addition of 0.4% urea.

^2SEM^, standard error of the mean.

^3^Lin, linear effect.

^4^Quad, quadratic effect.

^5^Cub, cubic effect.

### 3.5 Effect of urea on the relative abundance (%) of the affected bacteria at the genus level of cotton strawlage

Among all genera, 10 genera were affected by urea and presented in Table 7. The relative abundances of *Schleiferilactobacillus, Enterococcus*, and *Weissella* initially decreased and then increased with higher urea additions, peaking at 36.15% (U1), 1.36% (U2), and 1.54% (U3), respectively. Conversely, the trend of *Pantoea* was the opposite of these three genera. Three genera, including *Lactiplantibacillus, Aerococcus*, and *Massilia*, showed an increase with rising urea concentrations. Compared to U0, the relative abundances of *Pediococcus* and *Levilactobacillus* decreased across the other three treatments. The relative abundance of *Lacticaseibacillus* exhibited a trend of initially increasing, followed by a decrease again with greater urea addition.

**Table 7:** The relative abundance (%) of the affected bacteria at genus level of cotton strawlage after 45 days of fermentation with varying levels of urea.

**Phylum**	**Genus**	**Different levels of urea**				**SEM**	***P*-*value***
		**U0**	**U1**	**U2**	**U3**		
Firmicutes	*Schleiferilactobacillus*	5.92^b^	36.15^a^	5.29^b^	15.53^a^	4.604	0.039
	*Lacticaseibacillus*	3.80	8.40	1.20	10.63	1.724	0.068
	*Lactiplantibacillus*	2.25b^c^	0.94^c^	3.21^ab^	4.04^a^	0.362	0.003
	*Enterococcus*	0.49^b^	0.72^b^	1.36^a^	0.76^ab^	0.121	0.046
	*Weissella*	0.42^b^	0.48^b^	0.35^b^	1.54^a^	0.157	0.005
	*Aerococcus*	0.14^b^	0.46^ab^	0.62^ab^	0.87^a^	0.100	0.049
	*Pediococcus*	0.59^a^	0.20^b^	0.03^b^	0.05^b^	0.065	<0.001
	*Levilactobacillus*	0.17^ab^	0.04^b^	0.09^ab^	0.48^b^	0.073	0.033
Pseudomonadota	*Pantoea*	2.02^ab^	1.07^b^	1.12^b^	2.75^a^	0.243	0.021
	*Massilia*	0.02^b^	0.02^b^	0.04^b^	0.21^a^	0.028	0.03

^a − c^Values with different superscripts within the same row differ significantly (*P* < 0.05).

^1^U0, without urea; U1, the addition of 0.1% urea; U2, the addition of 0.2% urea; U3, the addition of 0.4% urea.

^2^SEM, standard error of the mean.

### 3.6 Correlation between affected bacteria and the urease and ammonia assimilation enzyme activities

**Figure 1:**
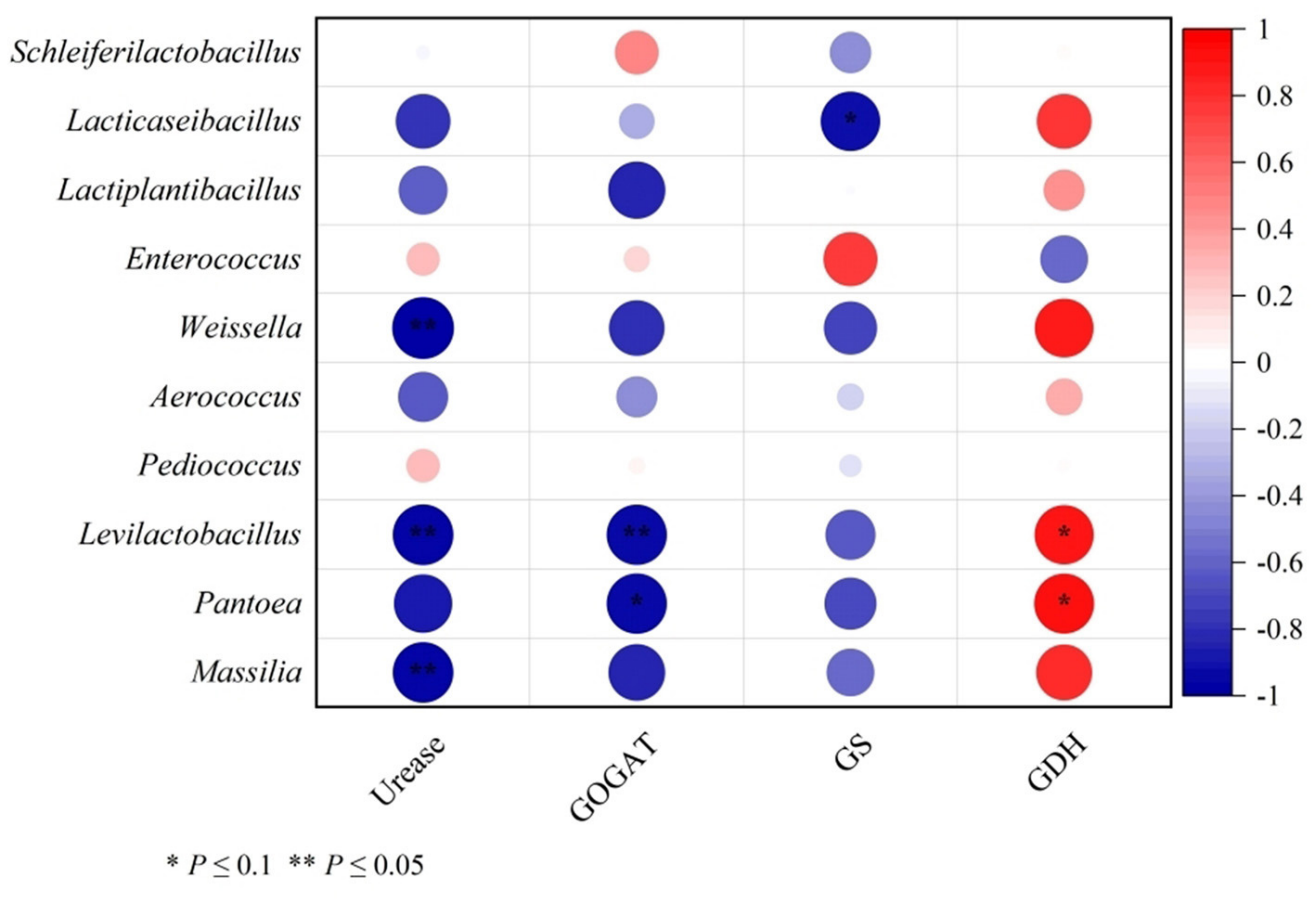
Correlation analysis of differential bacteria genus and urease, GOGAT, GS, and GDH concentrations of cotton strawlage after 45 days of fermentation with varying levels of urea. Each row represents a bacteria genus, only the genera with relative abundance > 0.01% are selected; each column represents an enzyme. The color blue means negative correlation, the color red means positive correlation. *P < 0.1, **P < 0.05.

Pearson correlation coefficients were calculated to study the correlation between various bacteria and the activities of urease, GOGAT, GS, and GDH. As shown in Figure 1, urease activity was negatively correlated with *Weissella, Levilactobacillus*, and *Massilia*. GOGAT displayed a negative correlation with the *Levilactobacillus* and tended to negatively correlate with the *Pantoea*. GDH tended to positively correlate with both *Levilactobacillus* and *Pantoea*.

### 3.7 Correlation between affected bacteria and the nitrogen distribution fractions

**Figure 2:**
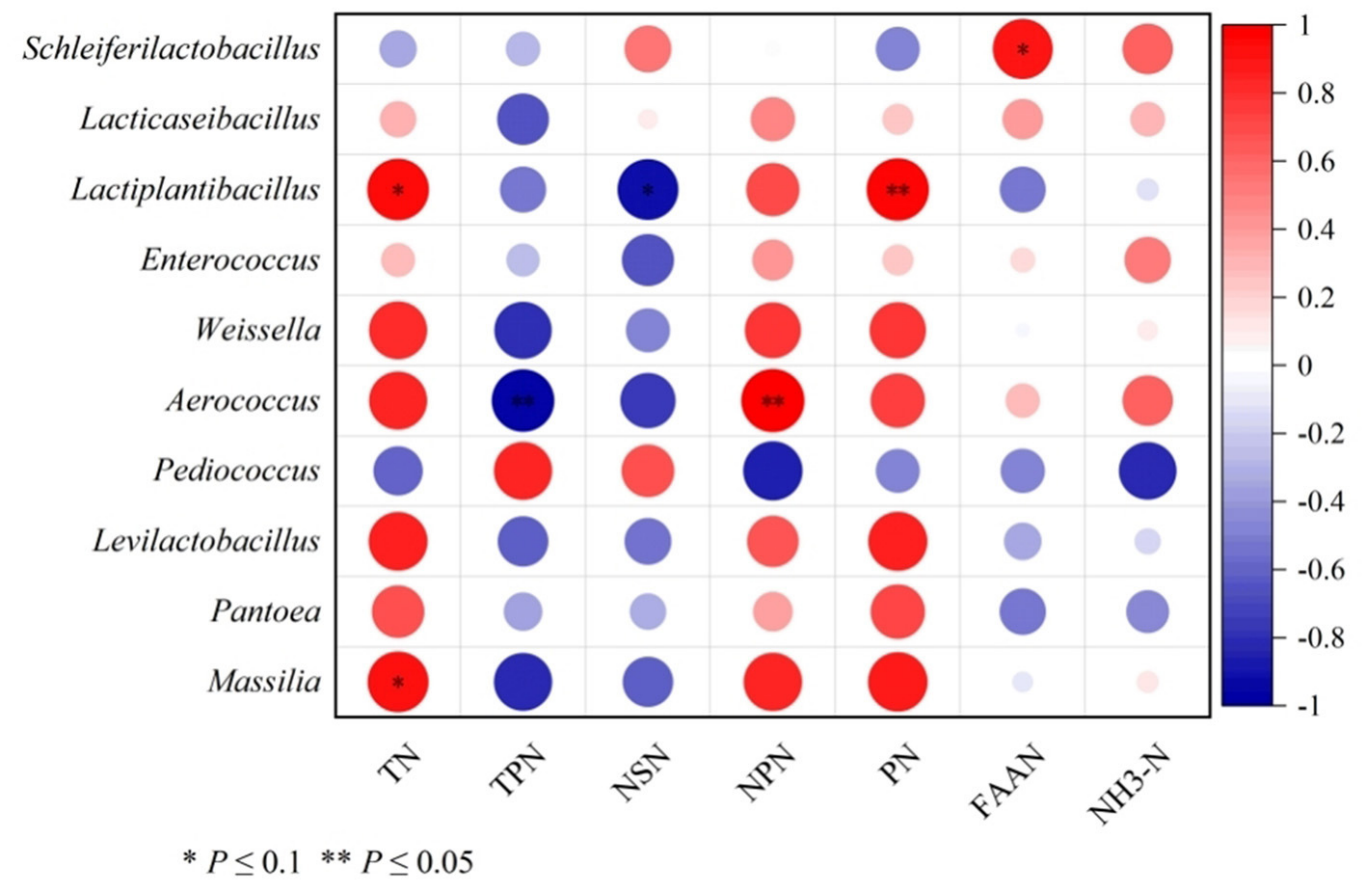
Correlation analysis of differential bacteria genera and nitrogen distribution fractions of cotton strawlage after 45 days of fermentation with varying levels of urea. Each row represents a bacteria genus, only the genera with relative abundance > 0.01% are selected; each column represents a nitrogen distribution fraction. The color blue means negative correlation, the color red means positive correlation. *P < 0.1, **P < 0.05.

As shown in Figure 2, TN content tended to positively correlate with *Lactiplantibacillus* and *Massilia*, while TPN was negatively correlated with the *Aerococcus*. Non-structural nitrogen (NSN) content tended to negatively correlate with the *Lactiplantibacillus*, while NPN content was positively correlated with the *Aerococcus*. PN content showed a positive correlation with *Lactiplantibacillus*, and FAAN content displayed a tendency to positively correlate with *Schleiferilactobacillus*.

### 3.8 Fermented metabolomics profiling

**Figure 3:**
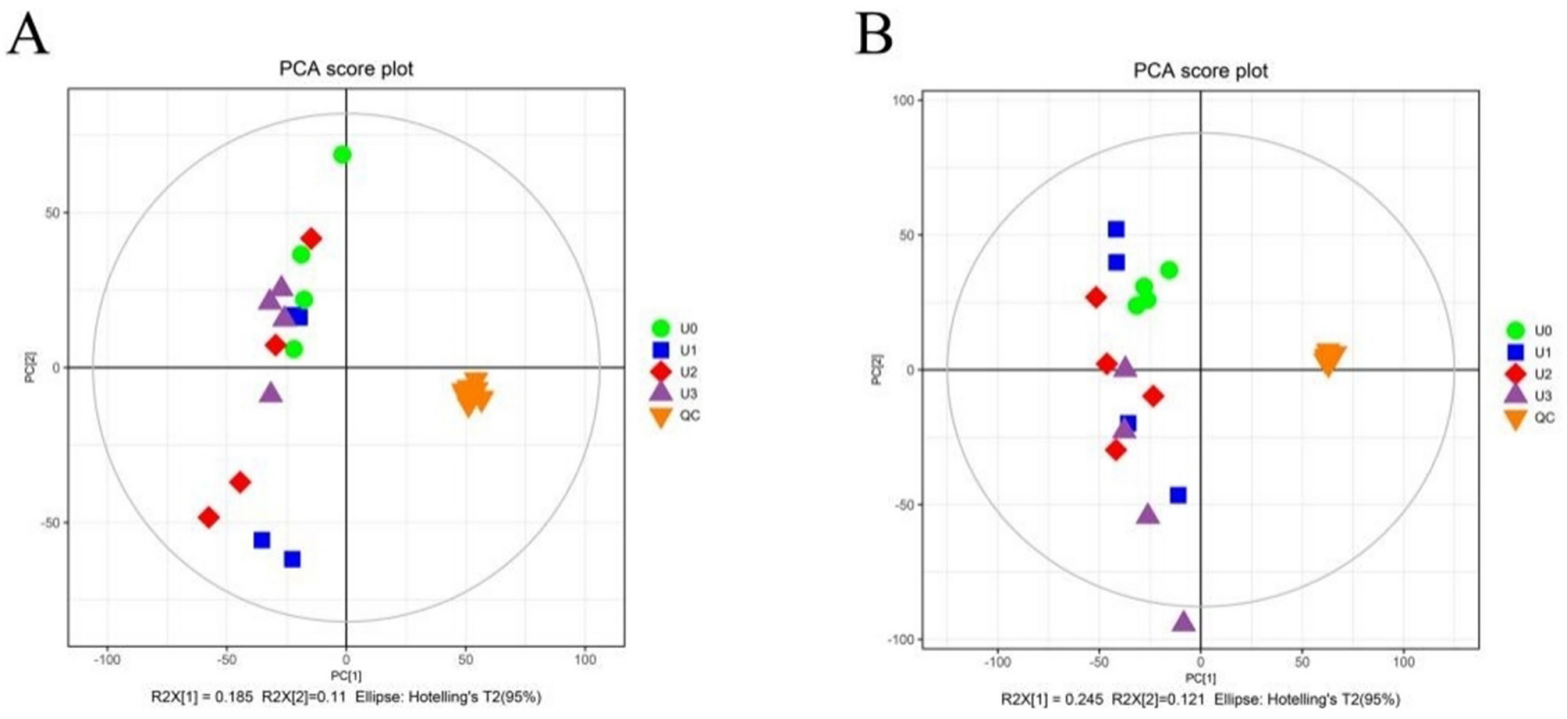
Principal component analysis (PCA) of cotton strawlage after 45 days of fermentation with varying levels of urea. **(A)** cationic mode; **(B)** anionic mode.

The principal component analysis (PCA) score plot was used to identify differences in metabolite data among the groups (Figure 3). The results indicate clear separation among samples from all groups. The OPLS-DA score plots validated the differentiated metabolites between each pair of groups, demonstrating distinct separation and discrimination under both positive and negative ion modes (Supplementary Figures S1, S2). The validation plots following the permutation test provided parameters for evaluating the OPLS-DA model's ability to distinguish among the four groups.

Volcano plot analysis visualized the effects of urea treatments on differential metabolites in fermented cotton straw (Supplementary Figure S3). In positive ion mode: U1 vs. U0 (Supplementary Figure S3a) identified 612 differential metabolites (161 down-regulated, 451 up-regulated); U2 vs. U0 (Supplementary Figure S3c) yielded 354 (207 down, 147 up); U3 vs. U0 (Supplementary Figure S3e) detected 801 (331 down, 470 up). In negative ion mode: U1 vs. U0 (Supplementary Figure S3b) identified 712 differential metabolites (302 down, 410 up); U2 vs. U0 (Supplementary Figure S3d) showed 542 (306 down, 236 up); U3 vs. U0 (Supplementary Figure S3f) revealed 1,016 (490 down, 526 up).

Metabolic pathway enrichment of the differential metabolites between U1, U2, U3, and U0 groups was visualized using topological bubble plots, as shown in Supplementary Figure S4. Given the complexity of metabolic responses, the top six pathways were selected for analysis based on their importance ranking. The pathways involved in U1 vs. U0 mainly included alanine, aspartate and glutamate metabolism, arginine biosynthesis, β-alanine metabolism, glyoxylate and dicarboxylate metabolism, arginine and proline metabolism, and pantothenate and CoA biosynthesis. The pathways for U2 vs. U0 comprised C5-branched dibasic acid metabolism, ubiquinone and terpenoid-quinone biosynthesis, tyrosine metabolism, β-alanine metabolism, arginine and proline metabolism, and arginine biosynthesis. U3 vs. U0 highlighted cutin, suberin and wax biosynthesis, glyoxylate and dicarboxylate metabolism, arginine and proline metabolism, arginine biosynthesis, ubiquinone and terpenoid-quinone biosynthesis, and the TCA cycle.

Based on VIP scores, fold changes (FC), and *P*-*value* (VIP > 1, FC > 1 or <1, *P* < 0.05), differential metabolites involved in the Top six metabolic pathways for each group were further screened (Supplementary Table S1). In the comparison of U1 vs. U0, six metabolites showed significant pathway-specific alterations: L-Proline, L-Alanine, and L-Glutamate were up-regulated, while L-Arginine, 3-Ureidopropionic acid, and Glyceric acid were down-regulated. In U2 vs. U0, seven down-regulated metabolites were identified: L-Proline, Citraconic acid, 4-Hydroxybenzoate, Homogentisic acid, 3-Ureidopropionic acid, L-Arginine, and 2-Oxoglutaric acid. The U3 vs. U0 comparison revealed 11 differential metabolites with L-Glutamate, L-Proline, Hydroxyproline, Citric acid, Glyceric acid, Glyoxylic acid, 2-Oxoglutaric acid, L-Arginine, L-Ornithine, and Homogentisic acid being down-regulated, except for 16-Hydroxyhexadecanoic acid, which was up-regulated.

## 4 Discussion

### 4.1 Impact of urea supplementation on nutritional characteristics of cotton strawlage

The DM content of the U2 treatment was lower than any other treatment, which was attributed to its high pH. Numerous studies have shown that high pH is not beneficial for DM preservation (Muck et al., 2018; Li et al., 2022). Saminathan, Wan Mohamed, Md Noh, Ibrahim, Fuat, and Kumari Ramiah (2022) study found that with increasing levels of urea inclusion (1%–5%), the contents of neutral detergent fibre (NDF) and acid detergent lignin (ADL) were significantly decreased. However, in this study, the ADF content of cotton strawlage did not continue to decrease with the increase in urea addition level. The study found that compared to the U0 treatment, 16-hydroxypalmitate was significantly upregulated in the cutin, suberin, and wax biosynthesis metabolic pathways of the U3 treatment. 16-Hydroxypalmitate serves as one of precursors for cutin biosynthesis (Li-Beisson et al., 2009). Increased cutin leads to the isolation of the plant surface from the surrounding environment (Chen J. Y. et al., 2023), thereby hindering the degradation of fibers by fiber-degrading bacteria and enzymes in the U3 treatment.

CP concentration serves as a pivotal determinant of feed nutritive value, directly correlating with dietary utility. In the current study, incremental urea supplementation in fermented cotton strawlage resulted in a statistically significant elevation in CP content relative to the U0 control group (*P* < 0.05), corroborating findings reported by Hou et al. (2022). Paradoxically, post-fermentation CP concentrations in all experimental groups (except U3) were lower than pre-fermentation values. This phenomenon may be attributed to incomplete suppression of aerobic microbial populations under suboptimal pH conditions, which perpetuated proteolytic activity and subsequent nitrogen loss (Hou et al., 2022; Shaani et al., 2017). WSC is a key factor in facilitating fermentation, serving as a microbial energy source during the micro-storage process. Xu (2022) also found that the content of WSC significantly affects the fermentation quality of cotton straw when adding different levels of molasses. A WSC content of 2%−3% DM is the threshold for continuous good fermentation and further reduction of pH (Hristov and Sandev, 1998). This well explains why the pH at the end of cotton straw fermentation in this study did not drop below 4.0.

The presence of free gossypol in cotton straw poses a significant limitation in their conversion to feed. Ruminants such as cattle and sheep can metabolize some free gossypol, but their metabolic capacity is limited. Prolonged feeding on diets high in cotton sources can lead to poisoning due to liver damage, posing serious health risks for the animals. In addition, excessive free gossypol intake can adversely affect the reproductive systems of male animals, leading to infertility (Hu et al., 2020; Wang et al., 2020). Studies have found that during the fermentation of cotton-based feed by microorganisms, specific enzymes produced by these microbes reduce the content of free gossypol in the feed (Wang et al., 2021, 2023; Ni et al., 2024). Urea serves as a nitrogen source for microbial growth and reproduction (Méndez-García et al., 2015; Hou et al., 2022). In this study, the gossypol degradation rate increased with the increase in urea addition levels, likely because a more abundant nitrogen supply promotes the proliferation of microorganisms and the production of gossypol-degrading enzymes—this relationship warrants further investigation. Additionally, based on the lack of a decline in gossypol degradation rate across the urea addition gradients tested in this experiment, we infer that the current urea levels have not yet reached the upper limit of microbial utilization. Second, Gossypol was noted as being less toxic to ruminants, and the current maximum level for free gossypol in complete feed for sheep, goat and cattle (except calves) is 300~500 mg/kg [EFSA Panel on Contaminants in the Food Chain (CONTAM) et al., 2017]. In this study, at the end of fermentation, the free gossypol content was less than the maximum level.

### 4.2 Impact of urea addition on fermentative characteristics of cotton straw

Li et al. (2021) and Wu et al. (2020) reported in their studies that Pseudomonadota and Firmicutes were the dominant phyla in fermented forage, and the phyla significantly affected by fermentation in this study were consistent with this finding. Most lactic acid bacteria (LAB) belong to the phylum Bacillota, which can increase lactic acid content in the system and rapidly create an acidic environment (Guan et al., 2018), further promoting the microbial composition to develop toward Firmicutes. pH, NH3-N, and volatile fatty acids are key indicators for preliminarily evaluating microbial fermentation of forage (Wang et al., 2020). Traditional ensiling practices suggest that forage with a post-fermentation pH below 4.2 is generally considered high-quality, but this threshold is closely associated with factors such as fermentation temperature, moisture content, and raw material characteristics (Ye et al., 2015; Wan et al., 2019; Wang et al., 2020; Shan et al., 2021). In this study, the pH values of groups U0 and U3 were lower than those of groups U1 and U2, which was due to the higher content of volatile fatty acids in these two groups. However, this study found that the relative abundance of LAB (lactic acid bacteria) was highest in group U1, particularly for ^*^Schleiferilactobacillus^*^, but its pH was not the lowest. This may be because: (1) The acid-producing efficiency of *Schleiferilactobacillus* is lower than that of genera such as *Lacticaseibacillus, Lactiplantibacillus*, and *Weissella*, but this requires further investigation to confirm; (2) The NH3-N content in group U1 was higher than that in groups U0 and U3, resulting in a higher pH.

### 4.3 Impacts of urea on the nitrogen fractions, urease activity, and ammonia assimilation enzyme activity of cotton straw

In the cotton straw fermentation process, efficient urea utilization requires the involvement of urea-decomposing bacteria, which synthesize urease to convert urea into ammonia nitrogen. This ammonia nitrogen can then be incorporated into microbial proteins through ammonia-assimilating enzymes such as GS, GOGAT, and GDH (Partow et al., 2017; Scarcia et al., 2017; Porcelli et al., 2018; Zhang et al., 2020; Cheng et al., 2022). In this experiment, GOGAT and GS activities were higher than that of GDH across all groups, indicating that ammonia assimilation primarily relied on GOGAT and GS. This explains why the total TPN content in the U3 treatment group was lower than in other groups. However, further analysis revealed significant negative correlations between GOGAT/GS activities and the abundances of *Levilactobacillus* and *Lacticaseibacillus*. Studies indicate that these two genera produce lactic acid to reduce silage pH (Chen Y. L. et al., 2023), suggesting that GOGAT and GS activities were inhibited in acidic environments. In contrast, GDH showed positive correlations with the abundances of most lactic acid bacteria and *Pantoea*, indicating that these microbes may preferentially utilize GDH for ammonia assimilation under low pH conditions. In this study, *Aerococcus* showed negative and positive correlations with TPN and NPN contents, respectively, indicating that *Aerococcus* may contribute to TPN loss and NPN accumulation. However, further research is needed to validate this finding. Studies have shown that *Schleiferilactobacillus* can degrade proteins into FAAN (Zheng et al., 2021), which aligns with the positive correlation observed between FAAN and *Schleiferilactobacillus* in this experiment. Additionally, a negative correlation between NSN content and lactate-utilizing bacteria was observed, particularly with a stronger negative correlation with *Lactiplantibacillus*. This effect may be due to lactate-utilizing bacteria breaking down the fibrous structure of cotton straw through organic acid production (Jia et al., 2013), and thereby degrading bound proteins. This is consistent with the results indicating NSN content in the U2 and U3 groups was significantly lower than that in the U0 and U1 groups.

### 4.4 Impact of urea on amino acids and metabolic profiles of cotton straw fermentation

PCA and OPLS-DA of metabolomics data from fermented cotton straw revealed significant separation between treatment groups with varying urea levels, indicating close correlations between metabolic profiles and urea addition. Existing studies have confirmed that energy metabolism, amino acid metabolism, and cofactor metabolic pathways play critical regulatory roles in forage fermentation processes, and significantly influencing fermentation quality (Du et al., 2021). This study further demonstrated that differential metabolites in fermented cotton straw were primarily enriched in amino acid metabolic pathways, with pathway complexity increasing significantly with higher urea levels. Notably, metabolites such as L-proline, L-arginine, L-glutamate, 3-ureidopropionic acid, glyceric acid, 2-Oxoglutaric acid, and homogentisic acid showed significant inter-group differences. Among these, L-proline and L-arginine showed consistent significant changes across all treatment groups, suggesting their central roles in urea-regulated metabolic networks of ensiled cotton straw. Pathway analysis indicated that variations in L-proline and L-arginine significantly impacted arginine biosynthesis and arginine/proline metabolic pathways. As a major component of collagen and milk proteins, proline is the amino acid with the highest demand for systemic protein synthesis (Wu et al., 2011) and play a role in maintaining cellular osmotic homeostasis (Christian, 1955). Proline biosynthesis occurs through two primary pathways: glutamate-dependent and ornithine-dependent routes (Fichman et al., 2015). This study found that compared to the U0 group, L-proline levels were significantly upregulated in the U1 group but downregulated in U2 and U3 groups (U3 <U2), consistent with amino acid quantification results. Possible mechanisms include: (1) the U1 group accumulated L-alanine and L-glutamate, providing sufficient substrates for proline synthesis; (2) the U2 group showed significantly reduced 2-Oxoglutaric acid, which may limit ornithine/glutamate production and inhibit proline synthesis through disruptions in energy metabolism. Additionally, coordinated downregulation of 2-Oxoglutaric acid, L-ornithine, and L-glutamate was observed in the U3 group, further supporting this hypothesis.

As a key amino acid for animal reproductive development and nutritional regulation (Sun et al., 2014; Fichman et al., 2015; Dubeibe et al., 2017), arginine biosynthesis relies primarily on glutamate-derived ornithine or citrulline precursors (Wang Q. et al., 2024). In this study, arginine levels were significantly lower in U1, U2, and U3 groups compared to U0, with decreasing amplitudes following the order U3 > U2 > U1. The reduced arginine levels in the U1 group may result from arginase-catalyzed decomposition into ornithine, which is subsequently converted to glutamate via ornithine aminotransferase (Liu et al., 2020), aligning with the observed upregulation of glutamate. The pronounced decreases in arginine in U2 and U3 groups likely stem from precursor ornithine deficiency, with reductions in 2-Oxoglutaric acid further evidencing the impact of energy metabolism on arginine synthesis.

## 5 Conclusion

The addition of 0.4% urea in ensiling cotton strawlage significantly increased the TN content, reduced the NSN and free gossypol contents, thereby enhancing the nutritional value of cotton strawlage, optimizing its nitrogen composition and mitigating feeding risks. In addition, the inclusion of urea in fermenting cotton strawlage significantly affected the composition of various bacterial genera. As urea addition levels increased, the amino acid and energy metabolism of cotton straw become increasingly complex.

## Data Availability

The original contributions presented in the study are publicly available. This data can be found here: https://www.ncbi.nlm.nih.gov, accession number PRJNA1237804.

## Supplementary material

Figure S1 Orthogonal partial least squares discriminant analysis (OPLS-DA) **(a, c, e)** and corresponding permutation test following positive mode ionization **(b, d, f)** derived from the metabolomics analysis of cotton strawlage after 45 days of fermentation with varying levels of urea. U0, without urea; U1, the addition of 0.1% urea; U2, the addition of 0.2% urea; U3, the addition of 0.4% urea.

Figure S2 Orthogonal partial least squares discriminant analysis (OPLS-DA) **(a, c, e)** and corresponding permutation test following negative mode ionization **(b, d, f)** derived from the metabolomics analysis of cotton strawlage after 45 days of fermentation with varying levels of urea. U0, without urea; U1, the addition of 0.1% urea; U2, the addition of 0.2% urea; U3, the addition of 0.4% urea.

Figure S3 Volcano plot analysis of differential metabolites in cotton strawlage after 45 days of fermentation with varying levels of urea. In the volcano plot, each dot represents a metabolite. The x-axis indicates the log2-transformed fold change of metabolites between groups, while the y-axis represents the -log10 transformed *P*-*value* from *t*-tests. The size of each dot corresponds to the VIP score derived from the OPLS-DA model, where larger dots denote higher VIP values. The up-regulated metabolites are colored red, the down-regulated metabolites are colored blue, and non-significant metabolites are shown in gray. And **(a, c, e)** are under positive mode ionization; **(b, d, f)** are under negative mode ionization.

Figure S4 Top six KEGG pathway enrichment analysis of differential metabolites from U1 vs. U0 **(a)**, U2 vs. U0 **(b)** and U3 vs. U0 **(c)** of cotton strawlage after 45 days of fermentation with varying levels of urea. The color is to distinguish the enrichment significance (- ln *P*-*value*), the darker the color is, the more significantly the metabolic pathway is enriched. The x-axis indicates the *P*-*value*. A larger size dot indicates higher pathway enrichment.
