# Supplementary figures and images for: Study on the effects of urea addition on the fermentation quality, nitrogen metabolism, microbial community, and metabolic characteristics of cotton strawlage
Source: Front Microbiol. 2025 Jun 23;16:1610850. doi: 10.3389/fmicb.2025.1610850 (PMC12233161; doi:10.3389/fmicb.2025.1610850)

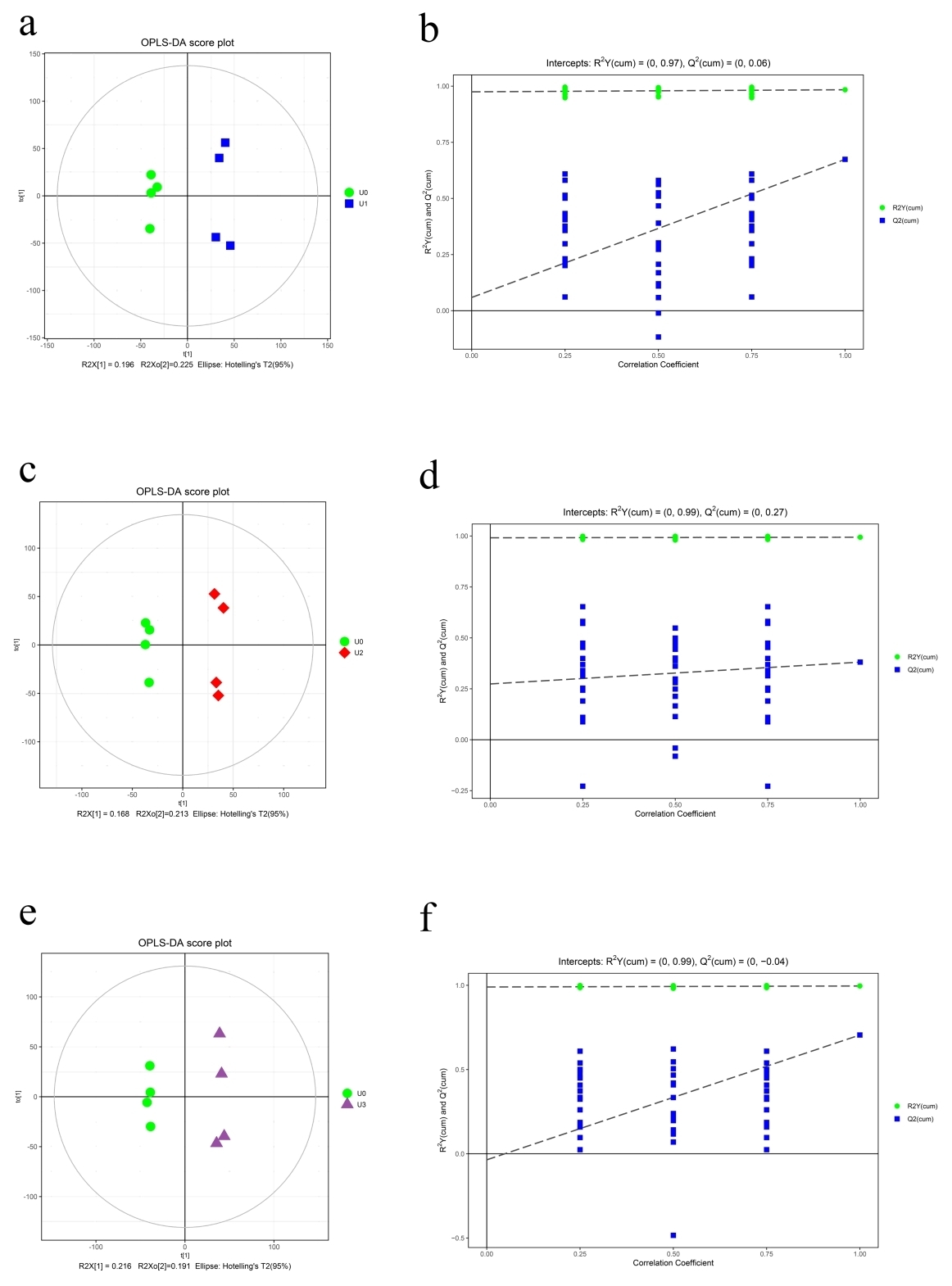

Supplement: Figure S1 — Orthogonal partial least squares discriminant analysis (OPLS-DA) (a, c, e) and corresponding permutation test following positive mode ionization (b, d, f) derived from the metabolomics analysis of cotton strawlage after 45 days of fermentation with varying levels of urea. U0, without urea; U1, the addition of 0.1% urea; U2, the addition of 0.2% urea; U3, the addition of 0.4% urea. [file Image_1.jpeg]

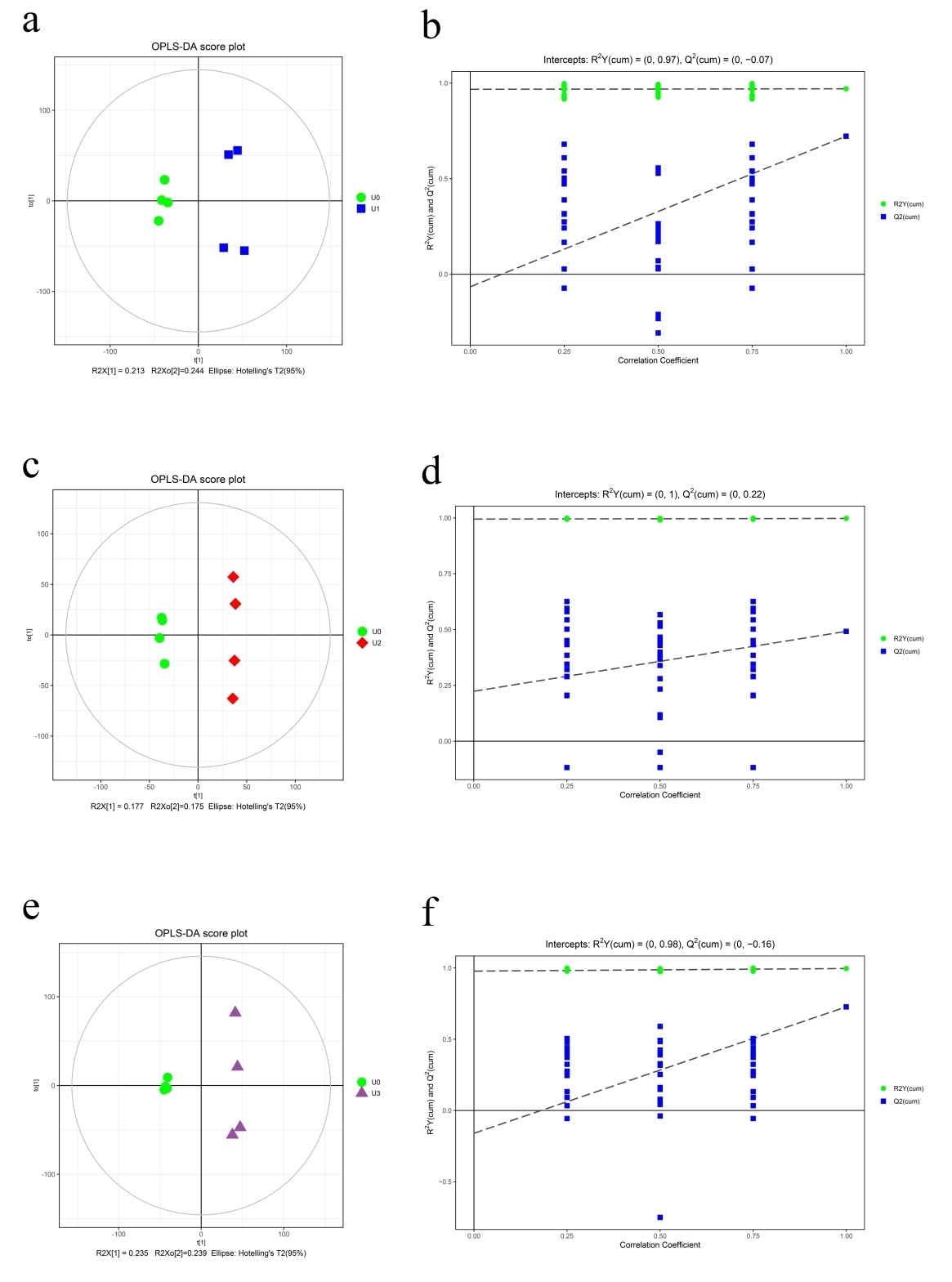

Supplement: Figure S2 — Orthogonal partial least squares discriminant analysis (OPLS-DA) (a, c, e) and corresponding permutation test following negative mode ionization (b, d, f) derived from the metabolomics analysis of cotton strawlage after 45 days of fermentation with varying levels of urea. U0, without urea; U1, the addition of 0.1% urea; U2, the addition of 0.2% urea; U3, the addition of 0.4% urea. [file Image_2.jpeg]

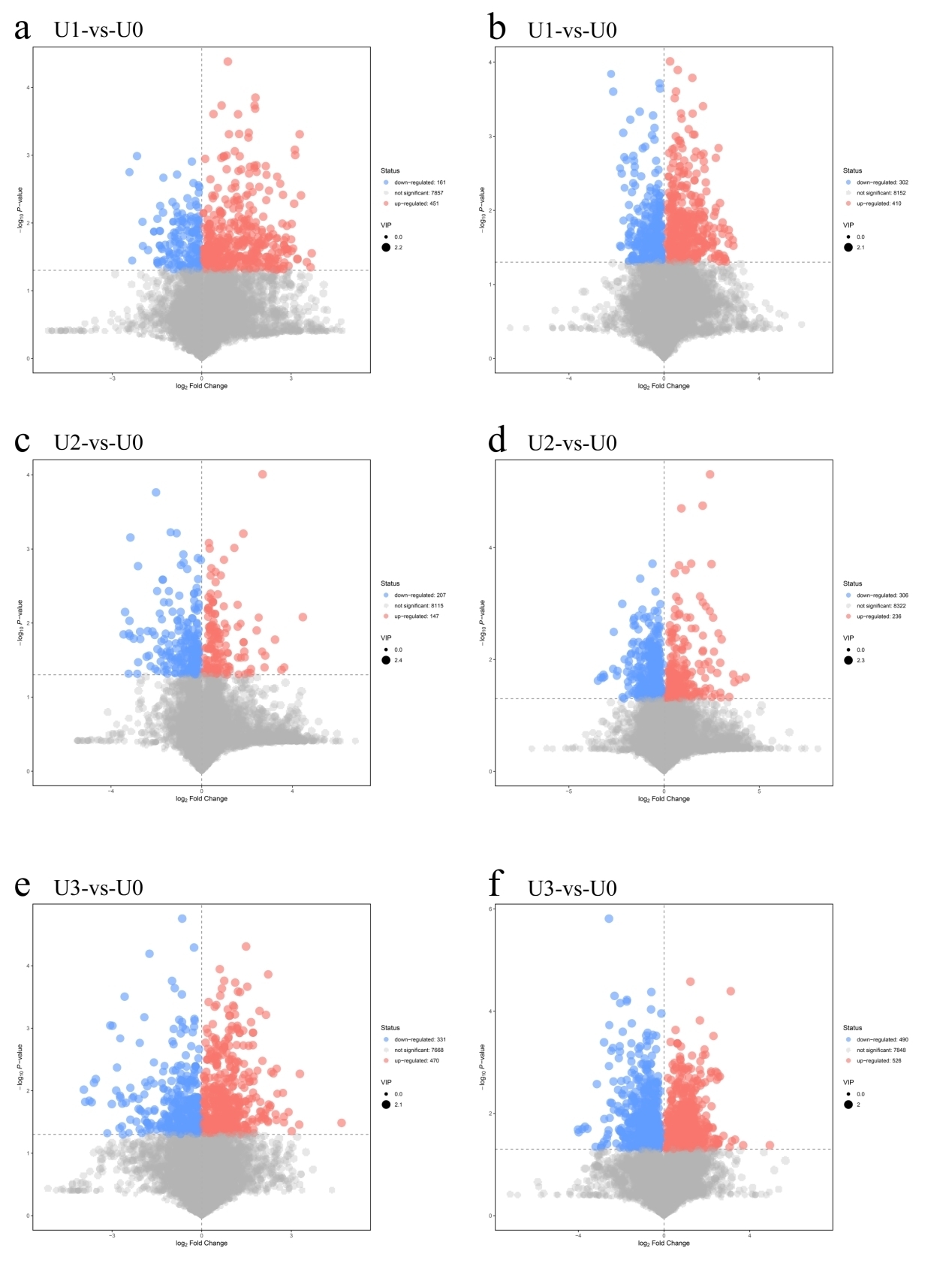

Supplement: Figure S3 — Volcano plot analysis of differential metabolites in cotton strawlage after 45 days of fermentation with varying levels of urea. In the volcano plot, each dot represents a metabolite. The x-axis indicates the log2-transformed fold change of metabolites between groups, while the y-axis represents the -log10 transformed P-value from t-tests. The size of each dot corresponds to the VIP score derived from the OPLS-DA model, where larger dots denote higher VIP values. The up-regulated metabolites are colored red, the down-regulated metabolites are colored blue, and non-significant metabolites are shown in gray. And (a, c, e) are under positive mode ionization; (b, d, f) are under negative mode ionization. [file Image_3.jpeg]

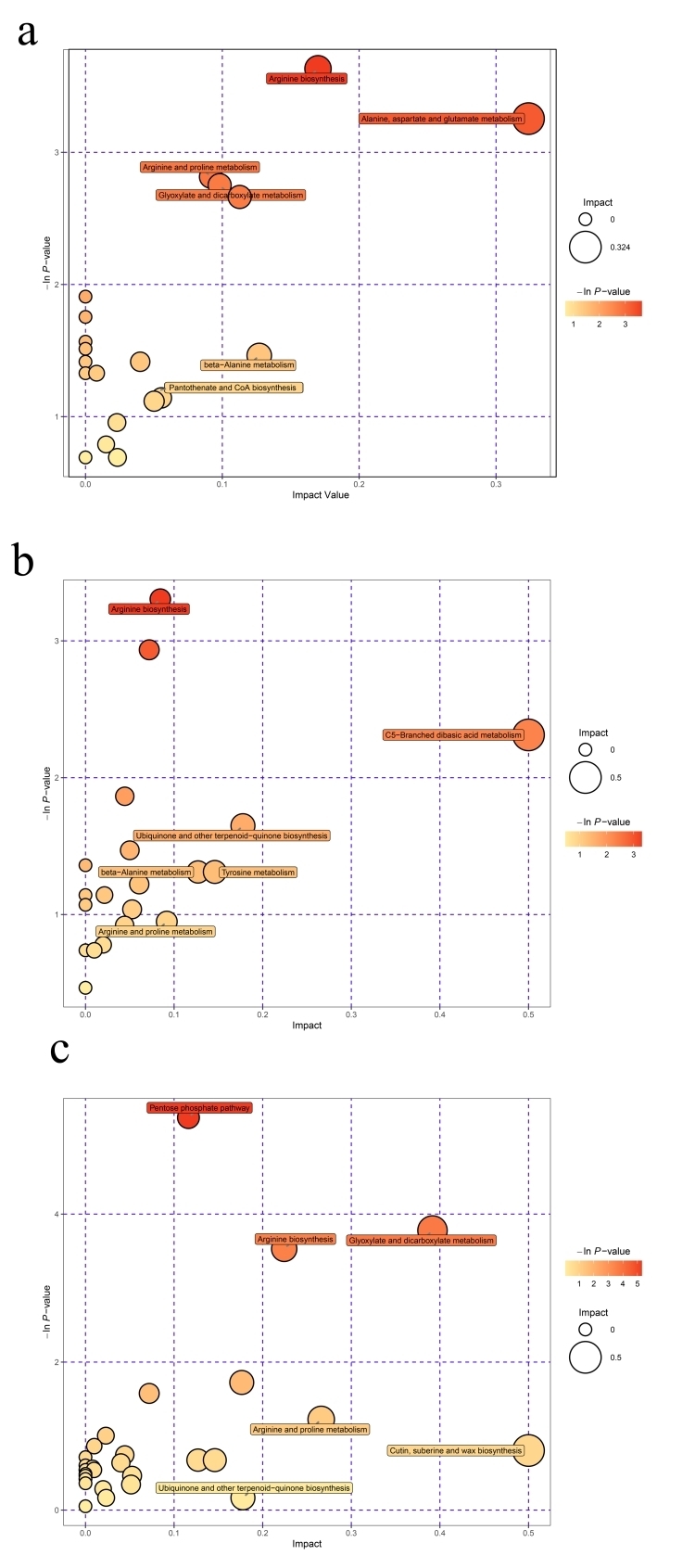

Supplement: Figure S4 — Top six KEGG pathway enrichment analysis of differential metabolites from U1 vs. U0 (a), U2 vs. U0 (b) and U3 vs. U0 (c) of cotton strawlage after 45 days of fermentation with varying levels of urea. The color is to distinguish the enrichment significance (- ln P-value), the darker the color is, the more significantly the metabolic pathway is enriched. The x-axis indicates the P-value. A larger size dot indicates higher pathway enrichment. [file Image_4.jpeg]
