# Supplementary material for: Study on the effects of urea addition on the fermentation quality, nitrogen metabolism, microbial community, and metabolic characteristics of cotton strawlage
Source: Front Microbiol. 2025 Jun 23;16:1610850. doi: 10.3389/fmicb.2025.1610850 (PMC12233161; doi:10.3389/fmicb.2025.1610850)
Supplement: Supplementary file 5 [file Table_1.docx]

Supplementary tables

| Table S1. Affected metabolites in the Top 6 pathways of U1 vs. U0, U2 vs. U0, and U3 vs. U0 of cotton strawlage after 45 days of fermentation with varying levels of urea | | | | |
| --- | --- | --- | --- | --- |
| Name | VIP^1^ | FC^2^ | *P*-value | Expression |
| U1 vs. U0 | | | | |
| L-Proline | 2.15 | 1.31 | 0.000 | up |
| L-Alanine | 1.79 | 1.16 | 0.033 | up |
| L-Glutamate | 1.64 | 1.26 | 0.004 | up |
| L-Arginine | 1.7 | 0.31 | 0.014 | down |
| 3-Ureidopropionate | 1.63 | 0.41 | 0.041 | down |
| Glyceric acid | 1.54 | 0.80 | 0.046 | down |
| U2 vs. U0 | | | | |
| L-Proline | 2.02 | 0.86 | 0.010 | down |
| Citraconic acid | 1.65 | 0.86 | 0.042 | down |
| 4-Hydroxybenzoate | 1.89 | 0.41 | 0.024 | down |
| Homogentisic acid | 2.04 | 0.54 | 0.006 | down |
| 3-Ureidopropionic acid | 2.22 | 0.11 | 0.019 | down |
| L-Arginine | 2.09 | 0.30 | 0.008 | down |
| 2-Oxoglutaric acid | 2.09 | 0.48 | 0.002 | down |
| U3 vs. U0 | | | | |
| L-Glutamate | 1.99 | 0.52 | 0.001 | down |
| L-Proline | 1.56 | 0.81 | 0.025 | down |
| Hydroxyproline | 1.72 | 0.67 | 0.014 | down |
| 16-Hydroxyhexadecanoic acid | 1.62 | 2.21 | 0.033 | up |
| Citric acid | 1.58 | 0.45 | 0.010 | down |
| Glyceric acid | 1.7 | 0.66 | 0.007 | down |
| Glyoxylic acid | 1.61 | 0.71 | 0.031 | down |
| 2-Oxoglutaric acid | 1.58 | 0.71 | 0.032 | down |
| L-Arginine | 1.91 | 0.11 | 0.003 | down |
| L-Ornithine | 1.79 | 0.63 | 0.010 | down |
| Homogentisic acid | 1.69 | 0.41 | 0.006 | down |
| ^1^VIP, variable importance in the projection of this substance from the OPLS-DA model for the group comparison.  ^2^FC, the fold change of this substance in the group comparison between the two experimental groups. | | | | |
